# Supplementary material for: Dynamic perceptual feature selectivity in primary somatosensory cortex upon reversal learning
Source: Nat Commun. 2020 Jun 26;11:3245. doi: 10.1038/s41467-020-17005-x (PMC7319990; doi:10.1038/s41467-020-17005-x)
Supplement: Supplementary file 1 — Supplementary Information [file 41467_2020_17005_MOESM1_ESM.pdf]

SUPPLEMENTARY MATERIAL

Dynamic perceptual feature selectivity in primary somatosensory cortex upon reversal learning

Ronan Chéreau et al.

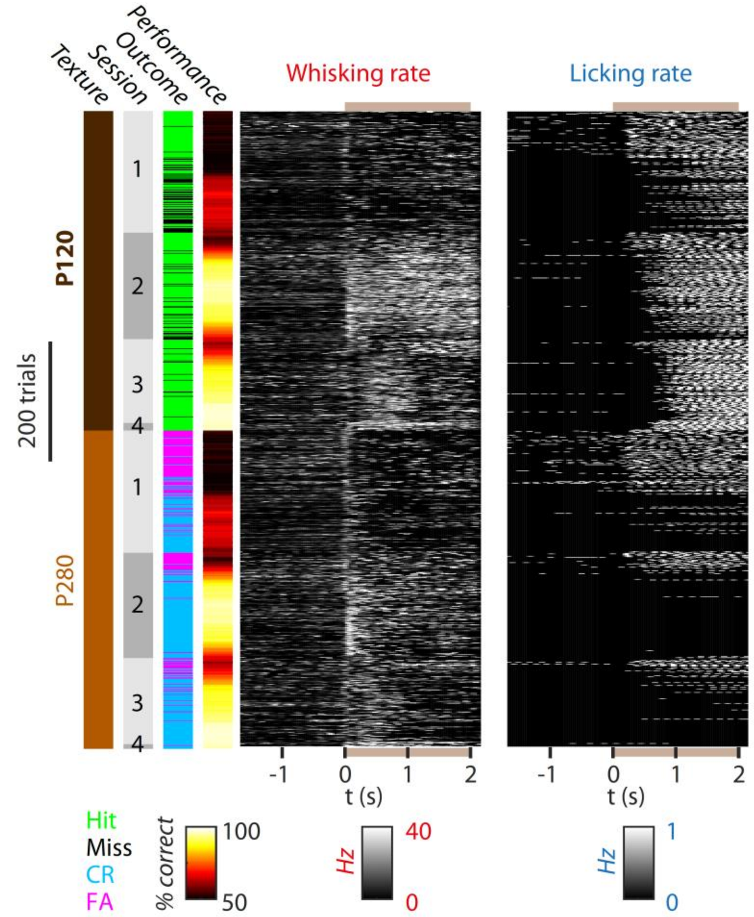

Supplementary Figure 1. Evolution of whisking and licking rates across learning.
Single trial whisking and licking rates for a mouse during training, aligned to texture presentation onset (*t*=0s). Trials are sorted by texture (P120 and P280 textures). For each trial, the corresponding session number, trial outcome and performance are shown. The shaded brown area indicates the time during which the texture was presented (0-2 s).

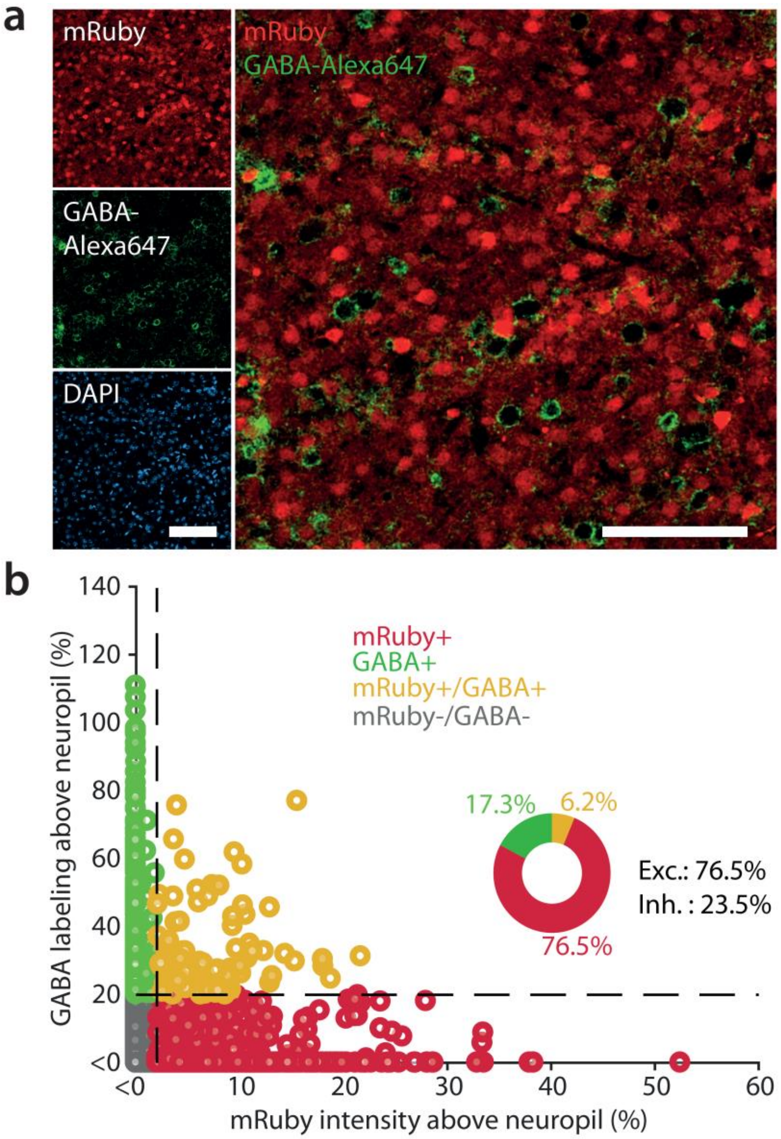

Supplementary Figure 2. Fraction of mRuby2-P2A-GCaMP6s-expressing neurons producing GABA.

a- Left, example of mRuby2-expression (red), GABA immunolabeling (green), and DAPI labeling (blue) of L2/3 neurons in S1. Right, a merge of the images showing mRuby2 expression and GABA immunolabeling. Note that the vast majority of mRuby-positive neurons is negative for GABA and vice versa. Scale bars: 100 μm. **b**- Scatter plot comparing mRuby2 fluorescence intensity to GABA immunolabeling fluorescence intensity both normalized to neuropil (each data point represents a neuron, *N*=2276 neurons measured from 6 mice used for training experiments but from different fields of views). A normalized signal intensity difference above 20% was considered as labeled. Pie chart shows the percentage of neurons that were mRuby2 positive only (red), GABA positive only (green), and mRuby2+GABA positive (yellow). Only 6.2% of mRuby2-positive neurons were GABA positive, indicating that the vast majority of mRuby2/GCaMP6s-expressing neurons are pyramidal neurons.

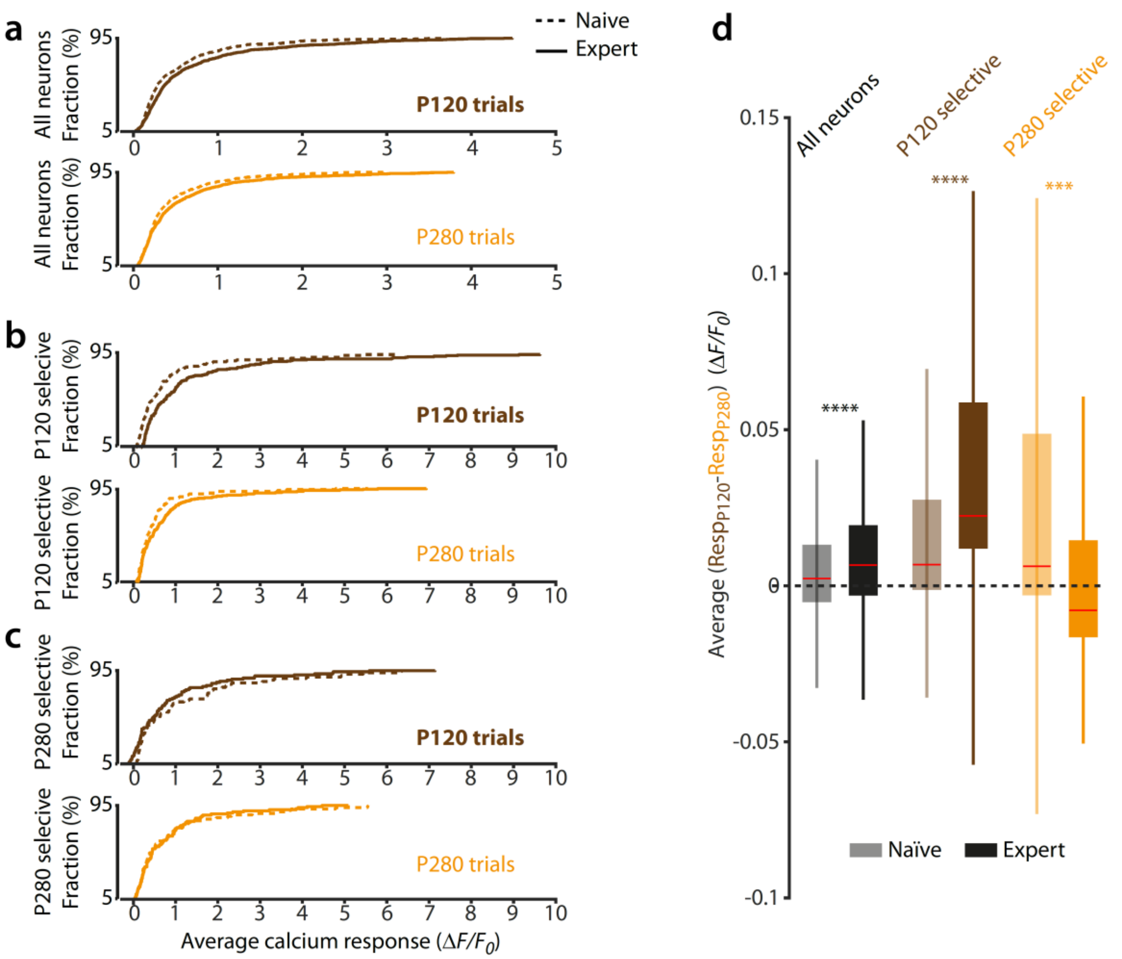

Supplementary Figure 3. Evolution of calcium signals across learning.

a- Cumulative distributions of the mean calcium signals per neuron for the P120 and P280 texture presentations in naïve (dashed line) and expert sessions (continuous line). For presentation purposes, the top and bottom 5% of the population measurements are not shown. **b**- Same for P120-selective neurons only. **c**- Same for P280-selective neurons only. **d**- Difference between normalized calcium signals in response to the P120 and P280 textures for all neurons (black), P120-selective neurons (brown), P280-selective neurons (orange), in naïve and expert sessions (all neurons: N=600 neurons in naïve and N=931 neurons in expert, Wilcoxon rank sum test naïve vs. expert, ***P=9x10^{-5}; P120-selective neurons: N=116 neurons in naïve and N=193 neurons in expert, Wilcoxon rank sum test naïve vs. expert, ***P=5.8x10^{-10}; P280-selective neurons: N=71 neurons in naïve and N=98 neurons in expert, Wilcoxon rank sum test naïve vs. expert, ***P=3.6x10^{-4}). Box plots show the median (red line), interquartile (box), and range (whiskers); outliers are not represented.

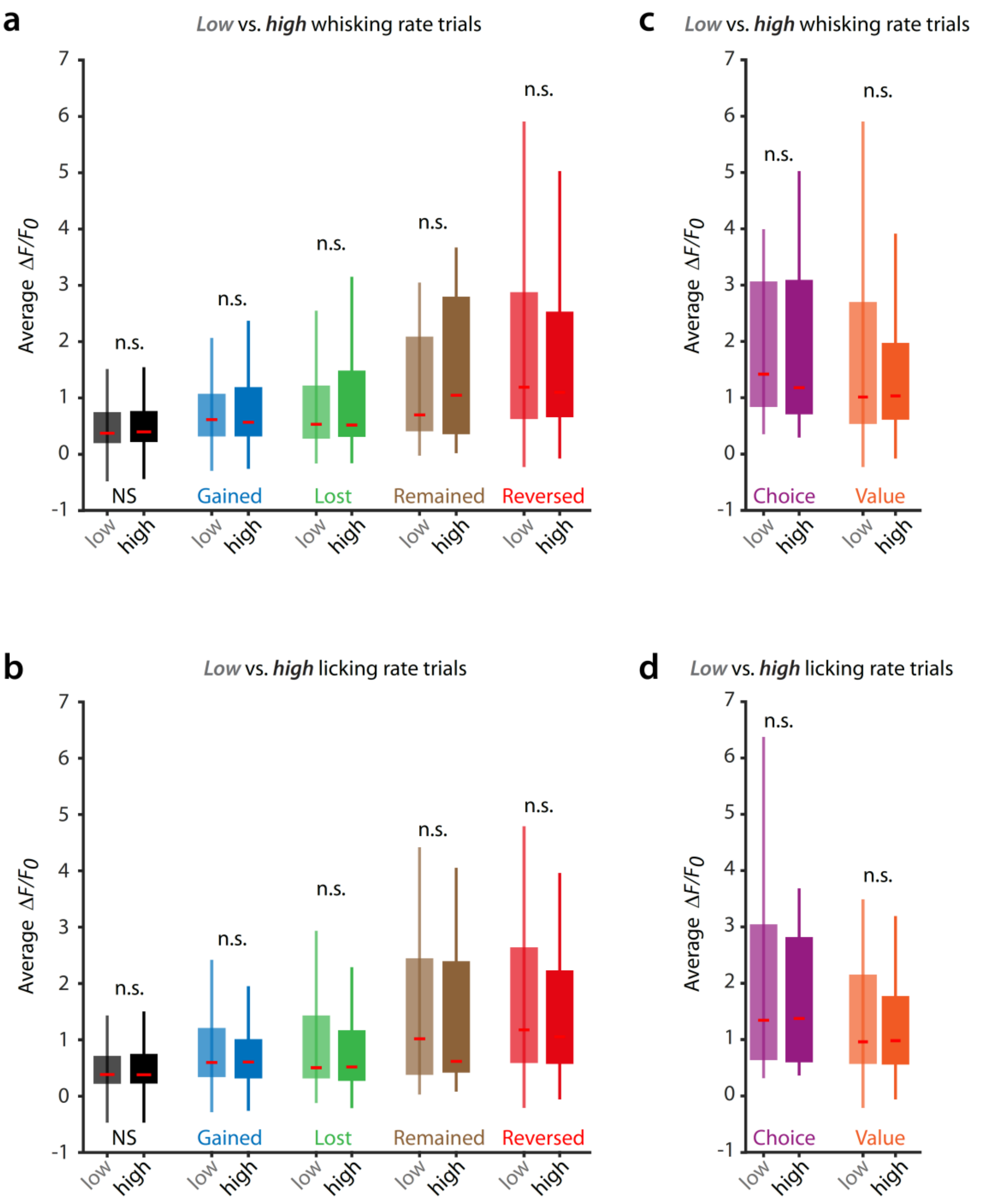

Supplementary Figure 4. Distributions of the average calcium signals in low vs. high whisking or licking rate trials for the identified classes of neurons.

a- Box plots represent the distribution of average calcium signals in each neuronal class for hit trials with low or high whisking rates (corresponding to the data presented in Fig. 4f). **b**- Same as (a) but for licking rates (corresponding to the data presented in Fig. 4g). **c**- Same as (a)but for value and choice neurons (corresponding to the data presented in Fig. 5d, top). **d**- Same as (c) but for licking rates (corresponding to the data presented in Fig. 5d, bottom). Box plots show the median (red line), interquartile (box), and range (whiskers); outliers are not represented.

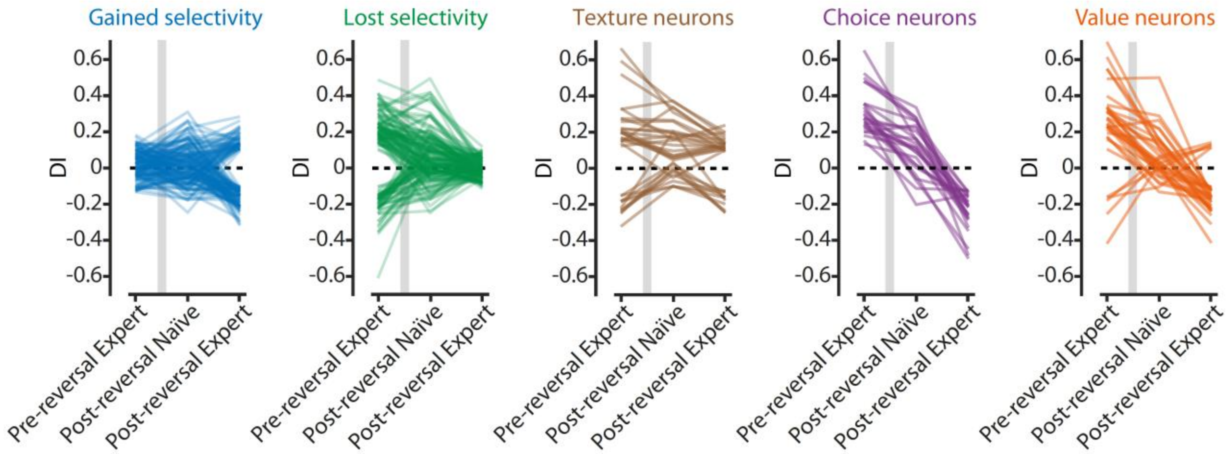

Supplementary Figure 5. The discrimination index for each neuronal class during reversal learning.

Evolution of DI of all neuronal classes in pre-reversal expert, post-reversal naïve, and post-reversal expert sessions (Neurons gaining selectivity after reversal, *N*=144; neurons losing selectivity after reversal, *N*=152; texture neurons, *N*=33; choice neurons, *N*=24; value neurons, *N*=35).

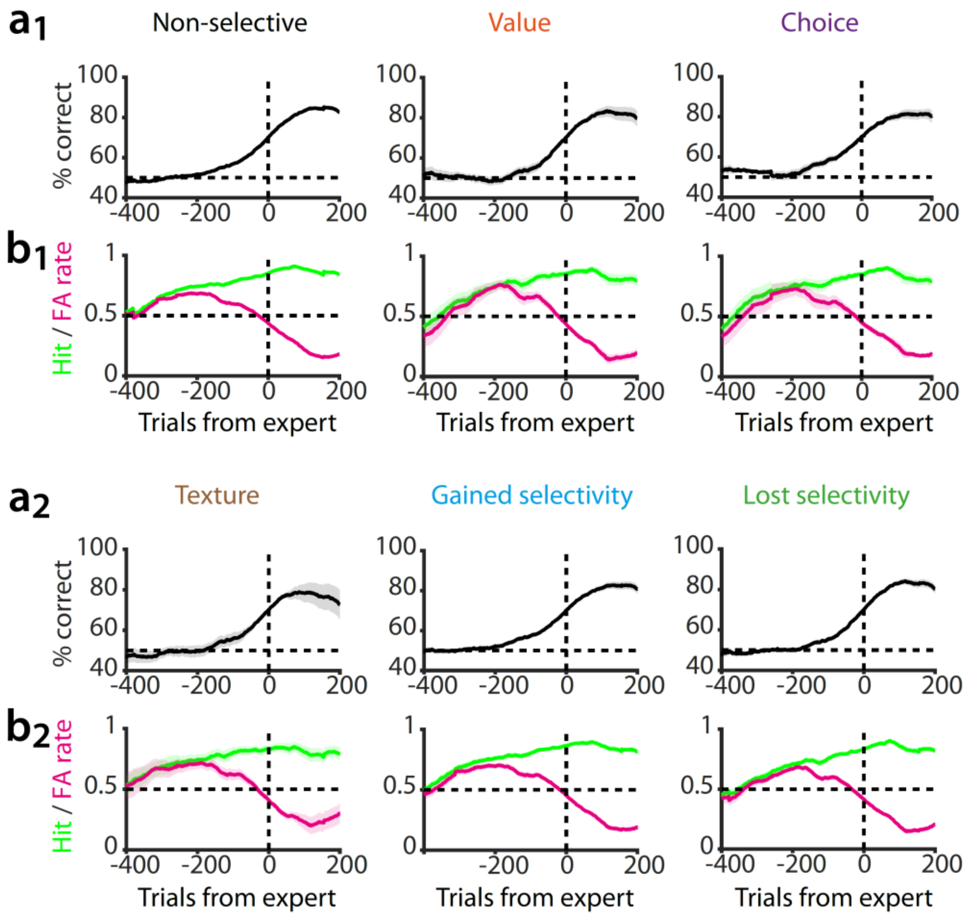

Supplementary Figure 6. Post-reversal performance of mice harboring each neuronal class.

a- All identified classes of neurons were not systematically detected in all mice. The average performance during reversal learning was calculated for the group of mice from which at least one neuron belonged to the given class. Performance curves were realigned to expert criterion (Non-selective neurons, *N*=412, 12 mice; Value neurons, *N*=35, 10 mice; Choice neurons, *N*=24, 6 mice; Texture neurons *N*=33, 7 mice, Neurons gaining selectivity after reversal *N*=144, 12 mice; Neurons losing selectivity after reversal *N*=152, 12 mice). **b**- Corresponding 'Hit' and 'FA' rates.

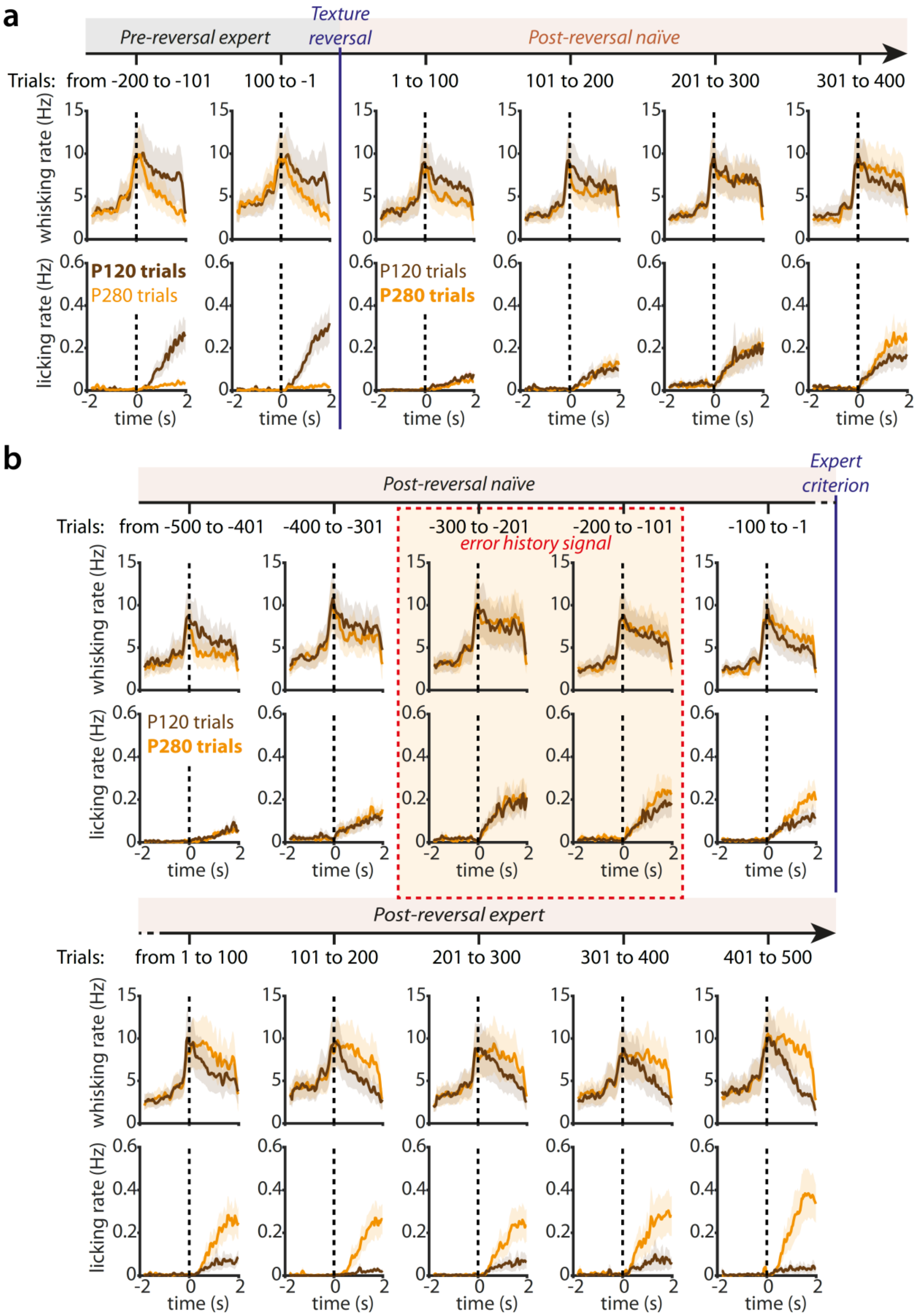
a
Pre-reversal expert
Texture reversal
Post-reversal naïve
Trials: from -200 to -101
100 to -1
1 to 100
101 to 200
201 to 300
301 to 400
whisking rate (Hz)
licking rate (Hz)
P120 trials
P280 trials
time (s)
b
Post-reversal naïve
Expert criterion
Trials: from -500 to -401
-400 to -301
-300 to -201
-200 to -101
-100 to -1
error history signal
Post-reversal expert
Trials: from 1 to 100
101 to 200
201 to 300
301 to 400
401 to 500

Supplementary Figure 7. Whisking and licking rates pre- and post-reversal learning.
a- Time course of whisking (top) and licking (bottom) rates aligned to texture presentation onset (dashed line) across two intervals of 100 trials before texture reversal (P120 texture was presented on the Go trials in green, P280 on the No-go trials in red) and the first four intervals after reversal (P280 texture was presented on the Go trials in green, P120 on the No-go trials in red). We observed a decrease in whisking and licking rates for the Go trials immediately after texture reversal. This may reflect a loss of commitment to execute the task, probably due to a decline in the number of water-rewards the mice receive as a result of an increased number of FAs and Misses. Whisking and licking rates gradually increase 200 trials after texture reversal when mice often start to randomly lick again upon presentation of the Go and No-go stimulus.
b- Average time course of whisking (top) and licking (bottom) rates aligned to texture presentation onset (dashed line) from five bins of 100 trials before and after the expert criterion (70% performance). The error history signal was detected in the value selective neurons within the -300 to -101 interval (dashed red area). Whisking and licking strategies only moderately changed during this time. When mice became experts, the whisking rates remained elevated upon the Go stimulus presentations, which may reflect whisker movements induced by the motor action of licking. Shaded areas represent SEM.

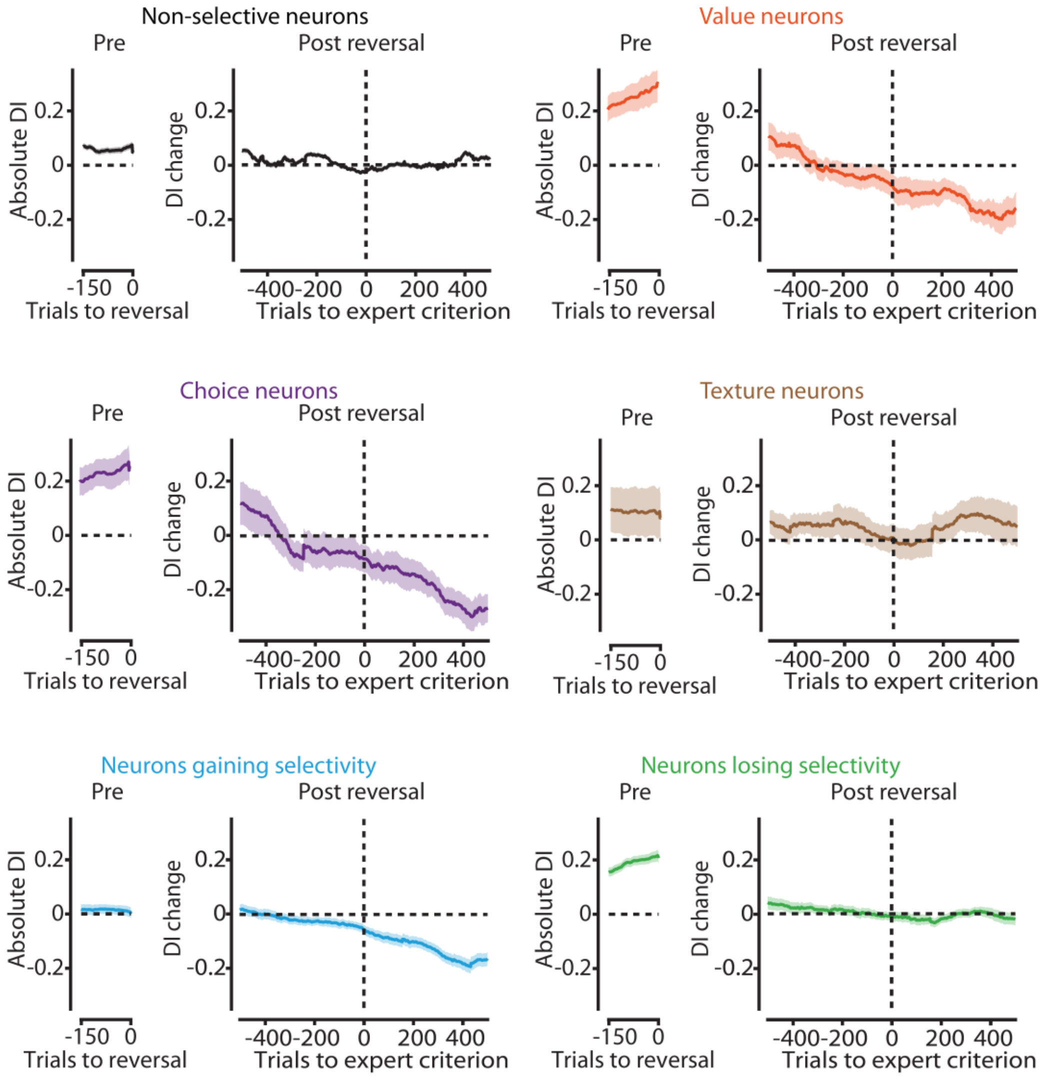

Supplementary Figure 8. Evolution of the discrimination index for each neuronal class.

For each panel, the graph on the left shows the average absolute DI across mice over the last 150 trials pre-reversal. The right graph shows the average change in DI from the absolute DI on the left, post-reversal, and realigned across mice to the expert criterion (Non-selective neurons, *N*=412, 12 mice; Value neurons, *N*=35, 10 mice; Choice neurons, *N*=24, 6 mice; Texture neurons, *N*=33, 7 mice, Neurons gaining selectivity after reversal, *N*=144, 12 mice; Neurons losing selectivity after reversal, *N*=152, 12 mice). Shaded regions represent SEM.

Mouse	Neurons recorded	Gained	Lost	Texture	Choice	Value	Non-selective
# 1	86	18	8	0	1	2	57
# 2	49	13	3	0	1	0	32
# 3	47	18	6	0	6	1	16
# 4	47	4	14	2	0	0	27
# 5	56	8	7	3	5	3	30
# 6	88	13	20	5	0	4	46
# 7	42	8	7	7	0	1	19
# 8	47	8	9	0	0	1	29
# 9	80	21	8	4	5	8	34
# 10	70	10	12	4	0	1	43
# 11	58	1	15	0	0	1	41
# 12	130	22	43	8	6	13	38
Total	800	144	152	33	24	35	412

Supplementary Table 1. Number of neurons in each class for all mice used in the training experiments.
