# Supplementary material for: Dynamic perceptual feature selectivity in primary somatosensory cortex upon reversal learning
Source: Nat Commun. 2020 Jun 26;11:3245. doi: 10.1038/s41467-020-17005-x (PMC7319990; doi:10.1038/s41467-020-17005-x)
Supplement: Supplementary file 3 — Reporting Summary [file 41467_2020_17005_MOESM3_ESM.pdf]

nature research

Corresponding author(s): *Double-blind peer review submissions: write DBPR and your manuscript number here instead of author names.*

Last updated by author(s): *YYYY-MM-DD*

Reporting Summary

Nature Research wishes to improve the reproducibility of the work that we publish. This form provides structure for consistency and transparency in reporting. For further information on Nature Research policies, see our Editorial Policies and the Editorial Policy Checklist.

Statistics

For all statistical analyses, confirm that the following items are present in the figure legend, table legend, main text, or Methods section.

n/a	Confirmed	
☐	☒	The exact sample size (n) for each experimental group/condition, given as a discrete number and unit of measurement
☐	☒	A statement on whether measurements were taken from distinct samples or whether the same sample was measured repeatedly
☐	☒	The statistical test(s) used AND whether they are one- or two-sided<br>*Only common tests should be described solely by name; describe more complex techniques in the Methods section.*
☐	☒	A description of all covariates tested
☐	☒	A description of any assumptions or corrections, such as tests of normality and adjustment for multiple comparisons
☐	☒	A full description of the statistical parameters including central tendency (e.g. means) or other basic estimates (e.g. regression coefficient) AND variation (e.g. standard deviation) or associated estimates of uncertainty (e.g. confidence intervals)
☐	☒	For null hypothesis testing, the test statistic (e.g. F, t, r) with confidence intervals, effect sizes, degrees of freedom and P value noted<br>*Give P values as exact values whenever suitable.*
☒	☐	For Bayesian analysis, information on the choice of priors and Markov chain Monte Carlo settings
☒	☐	For hierarchical and complex designs, identification of the appropriate level for tests and full reporting of outcomes
☐	☒	Estimates of effect sizes (e.g. Cohen's d, Pearson's r), indicating how they were calculated

Our web collection on statistics for biologists contains articles on many of the points above.

Software and code

Policy information about availability of computer code

Data collection	Scanimage 2016b was used to image neuronal activity (http://www.scanimage.org) running on MATLAB 2017a.<br>A custom-written LabWindows/CVI software (National Instruments) was used to record of the behavioral parameters.<br>VDaq software from Imager 3001F (Optical Imaging, Mountainside, NJ) was used to perform optical intrinsic imaging.
Data analysis	Custom-written scripts in MATLAB (version 2017a, Mathworks) and custom-written ImageJ (version 2.0.0; http://rsbweb.nih.gov/ij/) plugins were used to process the 2-photon images and custom-written scripts were used to analyse the behavior and calcium imaging data. Rigid lateral movement vectors were calculated using the NoRMCorre MATLAB toolbox (Mathworks). Winmix software from Imager 3001F (Optical Imaging, Mountainside, NJ) was used to analyse the optical intrinsic images.

For manuscripts utilizing custom algorithms or software that are central to the research but not yet described in published literature, software must be made available to editors and reviewers. We strongly encourage code deposition in a community repository (e.g. GitHub). See the Nature Research guidelines for submitting code & software for further information.

Data

Policy information about availability of data

All manuscripts must include a data availability statement. This statement should provide the following information, where applicable:

- Accession codes, unique identifiers, or web links for publicly available datasets
- A list of figures that have associated raw data
- A description of any restrictions on data availability

The data used to generate the figures is freely available at the CERN data repository Zenodo https://zenodo.org/communities/holtmaat-lab-data/ with DOI: https://doi.org/10.5281/zenodo.3824493. The principal Matlab code that was used for data analysis is freely available at the CERN data repository Zenodo https://zenodo.org/communities/holtmaat-lab-data/ with DOI: https://doi.org/10.5281/zenodo.3824493

Field-specific reporting

Please select the one below that is the best fit for your research. If you are not sure, read the appropriate sections before making your selection.

☒ Life sciences ☐ Behavioural & social sciences ☐ Ecological, evolutionary & environmental sciences

For a reference copy of the document with all sections, see nature.com/documents/nr-reporting-summary-flat.pdf

Life sciences study design

All studies must disclose on these points even when the disclosure is negative.

Sample size	We did not perform sample size calculations. Sample sizes were based on previous and similar types of studies (For example Huber et al 2012 Nature, Chen et al. 2015 Nature Neuroscience, Khan et al., 2018 Nature Neuroscience). These sample sizes are considered adequate and consistent with the existing literature.
Data exclusions	All acquired data were included for analysis, and are available in the data repository. None were excluded from the graphs, except in the distributions of Figure S3a-c and S4 some of the data points were not displayed for clarity purposes only. For the same reason, outliers were not displayed in the boxplots.
Replication	For the main data set several mice were trained consecutively in a texture discrimination task (maximally 4 mice were trained around the same time). One population of neurons was recorded per mouse in longitudinal fashion over the training period. We identified neuronal classes and categories posthoc, based on their activity patterns. All of the main classes and categories that were relevant for the study were found in most of the mice. For example, the neurons that are defined as value selective were found in 10 out of 12 mice (the remaining 2 mice didn't exhibit value selective neurons likely due to a limited neuronal population sampling). This has been specified in the manuscript (Supplementary Table 1). The whisker trimming experiment, in Fig. 1c, was repeated 7 times and we observed a drop in performance every time. The muscimol experiment, in Fig. 1d, was repeated 5 times and we observed a drop in performance every time.
Randomization	For the main data set, 6 week-old male mice were acquired from a commercial provider, and all received the same surgical procedures. All mice provided longitudinal data. Randomization was not required. For muscimol or trimming experiments, animals were randomly assigned to experimental groups without taking possible covariation into consideration. Data from the muscimol experiment were also used for another study, specified in the manuscript.
Blinding	The operators could not be blinded to training conditions, since all experiments were performed in longitudinal fashion, which could not be temporally randomized or permuted. In these regular training experiments, no assumption was made on the evolution of neuronal activity and therefore experimental blinding did not apply. For the trimming experiment, in Fig. 1c, and the muscimol experiment, in Fig. 1d, the operator could not be blinded because in both experiments, the intervention was performed by the experimenter and no vehicle controls were used. The effect of the intervention was readily visible by the operators (trimmed whiskers or injection hole through the cranial window for the muscimol experiment).

Reporting for specific materials, systems and methods

We require information from authors about some types of materials, experimental systems and methods used in many studies. Here, indicate whether each material, system or method listed is relevant to your study. If you are not sure if a list item applies to your research, read the appropriate section before selecting a response.

Materials & experimental systems

n/a	Involved in the study	
☐	☒	Antibodies
☒	☐	Eukaryotic cell lines
☒	☐	Palaeontology and archaeology
☐	☒	Animals and other organisms
☒	☐	Human research participants
☒	☐	Clinical data
☒	☐	Dual use research of concern

Methods

n/a	Involved in the study	
☒	☐	ChIP-seq
☒	☐	Flow cytometry
☒	☐	MRI-based neuroimaging

Antibodies

Antibodies used	mouse anti-GABA (ab86186, Abcam, Lot number: GR201297-3)<br>donkey anti-mouse antibody coupled to Alexa Fluor 647 (A32787, Thermo Fisher Scientific)
Validation	From manufacturer website:<br>Specificity ab86186 is highly specific to GABA and does not react with other amino acid BSA conjugates with the exception of weak reaction against beta alanine BSA conjugate at the highest concentrations used.

Tested applications Suitable for: IHC-P, ICC/IF, IHC-FoFr
Immunogen GABA coupled to BSA with glutaraldehyde

Animals and other organisms

Policy information about studies involving animals; ARRIVE guidelines recommended for reporting animal research

Laboratory animals	C57Bl/6J wild-type (males). Surgeries (start of the experiment) were done on 6 weeks old mice and behavioral and imaging experiments finished at 12-15 weeks-old. Animals were housed under standardized conditions in the animal facilities of the University of Geneva.
Wild animals	This study did not involve the use of wild animals.
Field-collected samples	This study did not involve the use of field collected samples.
Ethics oversight	All procedures were conducted in accordance with the guidelines of the Federal Food Safety and Veterinary Office of Switzerland and in agreement with the veterinary office of the Canton of Geneva (licence numbers GE/28/14, GE/61/17, and GE/74/18).

Note that full information on the approval of the study protocol must also be provided in the manuscript.
